# Patient and caregiver experiences with pantothenate kinase-associated neurodegeneration (PKAN): results from a patient community survey

**DOI:** 10.1186/s13023-023-02869-1

**Published:** 2023-08-31

**Authors:** Thomas Klopstock, Saadet Mercimek-Andrews, Agnieszka Jurecka, Patricia Wood, Maciej Cwyl, Angelika Klucken, Antonio López, Roberta Scalise, Andrea Valle, Fatemeh Mollet, Belen Perez-Duenas, Marta Skowronska, Magdalena Chroscinska-Krawczyk, Maria Luisa Escolar, Anna Wade, David Rintell

**Affiliations:** 1 Department of Neurology, Friedrich-Baur-Institute, LMU Klinikum, University Hospital of the Ludwig-Maximilians-Universität München, Munich, Germany; 2 Department of Medical Genetics, Faculty of Medicine and Dentistry, University of Alberta, Edmonton, Canada; 3 CoA Therapeutics, 1800 Owens Street, Suite C-1200, San Francisco, CA 94158 USA; 4 NBIA Disorders Association, El Cajon, USA; 5 Association NBIA Poland, Warszawa, Poland; 6 Hoffnungsbaum e.V., Würselen, Germany; 7 ENACH Asociación, Seville, Spain; 8 AISNAF, Rome, Italy; 9 FUERAN, Santo Domingo, Dominican Republic; 10 NBIA Suisse, Lausanne, Switzerland; 11 Department of Paediatric Neurology, Vall d`Hebron University Hospital, Barcelona, Spain; 12 Institute of Psychiatry and Neurology, Warsaw, Poland; 13 Department of Child Neurology, Medical University of Lublin, Lublin, Poland; 14 UPMC Children’s Hospital of Pittsburgh, Pittsburgh, CA USA

**Keywords:** Hallervorden–Spatz syndrome, NBIA, Neurodegeneration, Iron accumulation, Dystonia, PANK2, Atypical PKAN, Classic PKAN, Clinical outcomes assessment, Patient-oriented

## Abstract

**Background:**

Pantothenate kinase-associated neurodegeneration (PKAN) is a rare autosomal recessive genetic disorder of *PANK2*, which enables mitochondrial synthesis of coenzyme A. Its loss causes neurodegeneration with iron accumulation primarily in motor-related brain areas. Symptoms include dystonia, parkinsonism, and other disabilities. PKAN has been categorized as classic PKAN, with an age of onset ≤ 10 years, rapid progression, and early disability or death; and atypical PKAN, with later onset, slower progression, generally milder, and more diverse symptom manifestations. Available treatments are mostly palliative. Information on the lived experience of patients with PKAN and their caregivers or on community-level disease burden is limited. It is necessary to engage patients as partners to expand our understanding and improve clinical outcomes. This patient-oriented research study used multiple-choice and free-form question surveys distributed by patient organizations to collect information on the manifestations and disease burden of PKAN. It also assessed respondents’ experiences and preferences with clinical research to inform future clinical trials.

**Results:**

The analysis included 166 surveys. Most respondents (87%) were parents of a patient with PKAN and 7% were patients, with 80% from Europe and North America. The study cohort included 85 patients with classic PKAN (mean ± SD age of onset 4.4 ± 2.79 years), 65 with atypical PKAN (13.8 ± 4.79 years), and 16 identified as “not sure”. Respondents reported gait disturbances and dystonia most often in both groups, with 44% unable to walk. The classic PKAN group reported more speech, swallowing, and visual difficulties and more severe motor problems than the atypical PKAN group. Dystonia and speech/swallowing difficulties were reported as the most challenging symptoms. Most respondents reported using multiple medications, primarily anticonvulsants and antiparkinsonian drugs, and about half had participated in a clinical research study. Study participants reported the most difficulties with the physical exertion associated with imaging assessments and travel to assessment sites.

**Conclusions:**

The survey results support the dichotomy between classic and atypical PKAN that extends beyond the age of onset. Inclusion of patients as clinical research partners shows promise as a pathway to improving clinical trials and providing more efficacious PKAN therapies.

**Supplementary Information:**

The online version contains supplementary material available at 10.1186/s13023-023-02869-1.

## Background

Pantothenate kinase-associated neurodegeneration (PKAN) is a rare genetic disorder that affects an estimated 1–3 per 1,000,000 people worldwide [1, 2]. It is caused by autosomal recessive mutations of the *PANK2* gene, which encodes the form of pantothenate kinase critical for the synthesis of coenzyme A in mitochondria. Mitochondrial dysfunction and coenzyme A-mediated metabolic failure seem to trigger the neuropathology of PKAN, including iron accumulation resulting in injury to glia and GABAergic neurons in the basal ganglia and other brain areas and progressive neurodegeneration [3, 4]. Though rare overall and subject to broad regional variation, PKAN is one of the most common members of the group of disorders known as neurodegeneration with brain iron accumulation (NBIA), representing approximately one-third of the total documented cases of NBIA as of 2019 [5].

Because NBIA disorders cause degeneration in different brain areas and at different rates that are associated with local intracellular iron accumulation, symptoms and their expression over time can vary [2, 4]. The pattern of iron accumulation and neurodegeneration in PKAN is a diagnostic hallmark for the disease: iron accumulation in the basal ganglia creates a specific magnetic resonance imaging (MRI) pattern described as an “eye of the tiger” [6]. Expression of symptoms is similar between men and women and frequently begins in childhood. The main clinical features include extrapyramidal motor impairments, such as dystonia (involuntary muscle contractions), parkinsonism, and choreoathetosis (irregular involuntary limb and writhing movements), as well as pyramidal motor symptoms such as hyperreflexia and spasticity. Nonmotor symptoms can also occur and often present as visual impairment, cognitive decline, or developmental disabilities [2]. Typically, PKAN symptoms begin early in childhood, progression is rapid, and disability and/or death occur at a relatively early age (within about 20 years of symptom onset). However, atypical presentations of PKAN have been described in which disease onset occurs later, progression is slower, and symptom type/presence and severity are both highly variable [7].

The variety of identified PKAN phenotypes has driven the development of a putative classification system. Although still not officially accepted [8], patients with PKAN have been categorized as having classic or atypical PKAN differentiated primarily by the age of disease onset [1, 7]. Classic PKAN denotes the early-onset, rapidly progressing phenotype. Its hallmark features include onset of dystonia before the age of 10 years, loss of ambulation within 10–15 years of onset, and motor impairments that lead to early disability presenting as the primary symptoms (eg, overlapping dystonia-parkinsonism) [6, 7]. In atypical PKAN, dystonia onset presents after the age of 10 years, commonly in the second or third decade of life, and progression is usually much slower, such that loss of ambulation may occur between as many as 15 and 40 years after onset. Although the clinical features of atypical PKAN are significantly more varied than those of classic PKAN, motor impairment is still a primary symptom. However, its specific expression is more diverse and can include dysarthria or other motor difficulties [6, 7]. In addition, there is significant heterogeneity both within and between the phenotype classifications; descriptions of intermediate cases with early onset and slow progression, or late onset and fast progression, have been published [3]. Other aspects of PKAN that resist classification in this system include intrafamilial variability and evolution of a given patient’s phenotype over time. Despite this phenotypic diversity, classic and atypical PKAN generally exhibit differences in disease progression and symptom severity such that the less severe phenotypes are somewhat better associated with atypical PKAN [3, 7].

Whereas the biological and clinical features of PKAN have been described, much about the real-world experience of PKAN remains unclear, in large part due to the rarity of the disease. For example, there is limited information regarding the lived experience of patients and caregivers and almost no information on the disease burden of PKAN at a larger (eg, population-level) scale [8]. Equally scarce is literature describing the impact of PKAN on patients and family members. This information gap impedes the clinical research necessary to advance care and treatment of patients with PKAN. It is critical, therefore, to engage patient advocacy organizations (PAOs) and, when feasible, patients themselves as research partners. Such engagement can uniquely enable the gathering of information on patient-related factors in PKAN therapy to improve clinical trial design (eg, increase subject recruitment/retention and account for patient preferences), with the goal of improving the accuracy of trial results and ultimately providing patients with safer and more efficacious care and treatment.

The objective of the current study was to address this information gap through patient-oriented research, using surveys to collect data from patients with PKAN and their caregivers and families. These results will allow the description of the patient community experience with PKAN and inform best practices for future clinical trials. To accomplish this, we engaged PAOs to help develop and deploy a survey to their patient communities. Questions focused on disease manifestation and burden (both patient-level disease burden and effects on quality of life), disease management, and respondents’ experiences with and preferences for future clinical trials.

## Results

### Population characteristics

A total of 182 surveys were returned. Of these, 16 were disqualified for various reasons, most often because the respondent incorporated more than one patient’s responses into a single survey; the remaining 166 were included in the analysis. The majority of respondents (87%; n = 145) were parents of a patient with PKAN, 7% (n = 11) identified as patients, 5% (n = 9) identified as “other caregivers” of a patient, and one respondent identified as someone assisting a patient or caregiver. Descriptive analyses of the responses were conducted on the whole dataset and on each of the classic and atypical PKAN groups. No comparative between-group tests for statistical significance were performed, but notable differences (defined as a ≥ 10% difference in a value between groups) were identified. The vast majority of respondents were from North America and Europe (80%), with the remaining respondents representing a variety of other countries. One respondent did not provide a specific country, instead responding with “Other”. The majority of patients (66%; n = 110) were 12–29 years of age, and 18% were < 12 years of age at the time of survey response (Table 1). Using a cutoff of 10 years old at symptom onset) [2], the sample was grouped into patients with classic PKAN (n = 85) and atypical PKAN (n = 65), with 16 individuals who were not sure when symptoms started classified as “not sure.”

**Table 1:** Population Characteristics of Respondents for the 166 Surveys Included in the Study

Characteristic	No. of Respondents
*Self identification, n (%)*	
Parent of patient	145 (87%)
Patient	11 (7%)
Other caregiver*	9 (5%)
*Location: Europe, n*	
Poland	34
Germany	15
Italy	15
Spain	12
Slovenia	4
United Kingdom	3
Austria	3
*Location: the Americas, n*	
Dominican Republic	27
United States	24
Brazil	4
Canada	4
Mexico	3
*Location: other region, n*	
India	3
Other nation	1 each: the Netherlands, Switzerland, Cuba, Ukraine, Saudi Arabia, Norway, Egypt, Iran, New Zealand, Jordan, France, Other**
*Age at survey response, y, n (%)*	
< 12	30 (18%)
12–17	49 (30%)
18–29	61 (37%)
≥ 30	26 (16%)

*1 respondent self-identified as someone assisting a patient/caregiver

**1 respondent identified their country of origin only as “other”

### Disease presentation and clinical manifestations

**Fig. 1:**
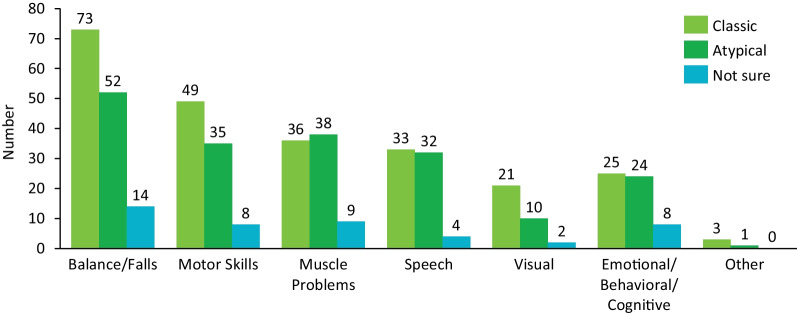
First symptoms experienced by respondents according to PKAN phenotype. Respondents (N = 166) were grouped into classic (pink, n = 85) and atypical (green, n = 65) PKAN phenotype groups based on their reported age of onset (classic, < 10 years). Respondents who were unable to provide an age of onset were grouped as “not sure” (purple, n = 16). PKAN = pantothenate kinase-associated neurodegeneration

**Fig. 2:**
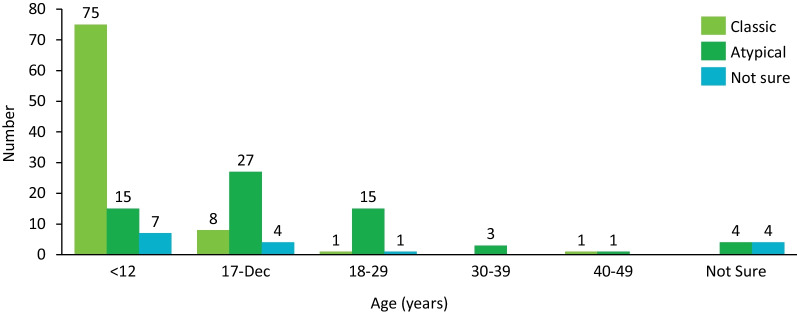
Respondent age at diagnosis (years) according to PKAN phenotype. Respondents were grouped into classic (pink) and atypical (green) PKAN phenotype groups based on their reported age of onset (classic, < 10 years). Respondents who were unable to provide an age of onset were grouped as “not sure” (purple, n = 16). PKAN = pantothenate kinase-associated neurodegeneration

Regardless of PKAN phenotype groups, respondents overall most frequently reported their first experienced symptoms (Fig. 1) to be problems with balance or falling, poor motor skills (sometimes perceived as clumsiness), and/or problems with muscle function (eg, cramping, postural problems). Notable differences between groups in terms of first symptoms experienced emerged and included muscle problems (classic, 42.4%; atypical, 58.5%), speech difficulties (classic, 38.8%; atypical, 49.2%), and visual problems (classic, 24.7%; atypical, 15.4%). Respondents in the classic PKAN group reported an age of diagnosis under 12 years 88.2% of the time, and only 2.4% received a diagnosis at 18 years or older, whereas 23.1% of respondents in the atypical PKAN group received a diagnosis at age 12 years or earlier, and 35.4% at age 18 years or older (Fig. 2). The mean ± SD age of onset for the classic PKAN group was 4.4 ± 2.79 years and the median was 4 years (range, 0.1–9.5 years); that for the atypical group was 13.8 ± 4.79 years and the median was 13 years (range, 10–35 years).

**Fig. 3:**
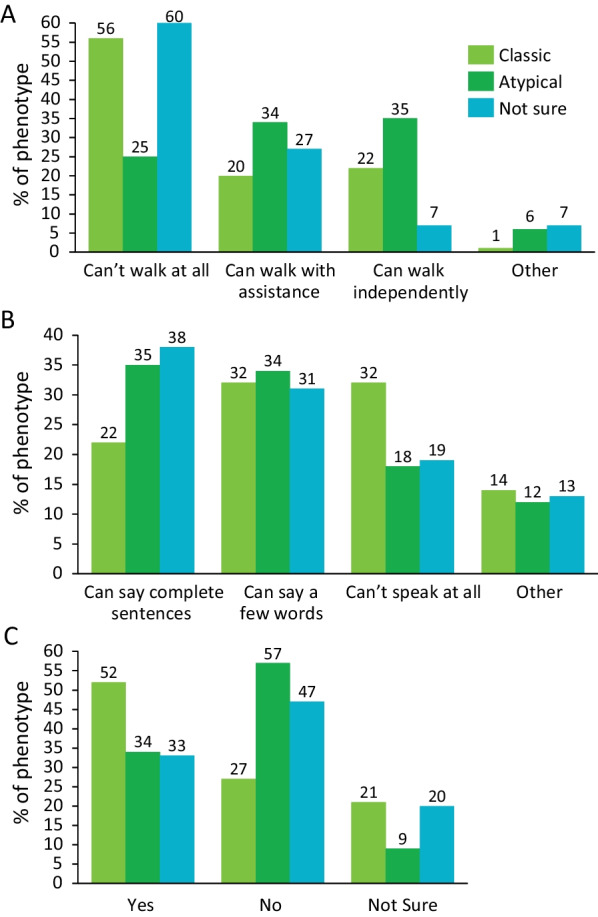
Most pronounced symptom manifestations. Respondents (N = 166) described 3 broad categories of symptom manifestations: motor/walking (**A**), speech (**B**), and visual (**C**) impairments. Responses included either a complete loss of function or loss of some level of capability (below full functionality). PKAN = pantothenate kinase-associated neurodegeneration

When reporting on the symptoms they experienced, respondents in both groups reported motor difficulties, usually expressed as gait disturbances, most often. Patients with atypical PKAN whose age of symptom onset was in their second decade of life or later often reported parkinsonian symptoms (eg, a slowed or frozen walk) as the disease progressed. In these patients, other motor problems presented as difficulty with speech, while visual problems were also common. Overall, 44% of the patients were unable to walk, 26% were unable to speak, and 43% had difficulty seeing (Fig. 3). Motor difficulties were more severe in the classic PKAN group, with 51.8% reporting a complete loss of ambulation (vs. 21.5% in the atypical group), and 20% of the respondents with classic PKAN reporting being unable to sit (vs. 3.1% in the atypical group). A substantial number of respondents with classic PKAN (36.9%) reported needing a feeding tube due to swallowing difficulties. A similar pattern between classic and atypical PKAN was found when examining speech (32% of the classic PKAN group and 18% of the atypical PKAN group reported a loss of speaking ability) and visual (44% of the classic PKAN group and 34% of the atypical group) difficulties.

### Disease burden and management

**Fig. 4:**
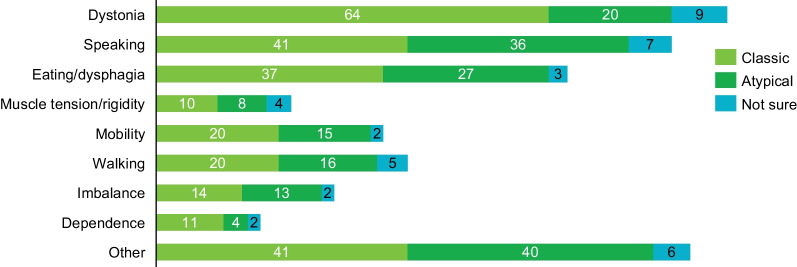
Most challenging symptoms according to presenting PKAN phenotype. Respondents were grouped into classic (blue) and atypical (dark green) phenotypes based on age of diagnosis. All respondents provided the top 3 symptoms they felt were most challenging to them. The numbers within the bars represent the number of times that symptom was mentioned in total by that phenotype group. PKAN = pantothenate kinase-associated neurodegeneration

When asked to describe their experiences related to the disease burden of PKAN, respondents in both groups ranked dystonia, speech problems, and difficulties with eating and swallowing as the most challenging symptoms (Fig. 4). Many of the other symptoms that patients with PKAN reported as most challenging were related to motor dysfunction (eg, walking and muscle rigidity). Disease burden was not exclusively physical, as over 10% of the responses mentioned the lack of independence or social skills and other behavioral or cognitive symptoms.

Respondents were also asked to consider the top 3 symptoms they hoped to be improved by new treatments. These rankings mostly corresponded with those regarding the disease burden patients reported experiencing. However, speaking and speech problems ranked highest (90 mentions) when patients were asked about their most desired symptom relief, whereas dystonia ranked highest (84 mentions) when asked about their most burdensome symptoms.

Respondents were asked to describe any interventions used to manage their disease and were allowed to select as many as they felt applied to them. The majority of respondents reported using 2 or more interventions, primarily multiple medications and/or wellness products such as over the counter vitamins and plant- and animal-derived nutritional supplements. A total of 509 specific medications and 406 wellness products were reported (see Additional files 1: Table S1 and 2: Table S2). Among medications, anticonvulsants (146 specific drug mentions), antiparkinsonians (97 mentions), and skeletal muscle relaxants (96 mentions) were most frequently mentioned drug classes. Wellness products mentioned most often fell into the categories of vitamin B (83 mentions), fatty acids (56 mentions), and vitamin D (50 mentions). Only 13.8% of respondents reported taking no medications. Other specific interventions reported in relatively high numbers included the use of feeding tubes (26% of patients with PKAN), deep-brain stimulators (24%), and botulinum toxin (28%).

### Clinical trial experiences and preferences

**Fig. 5:**
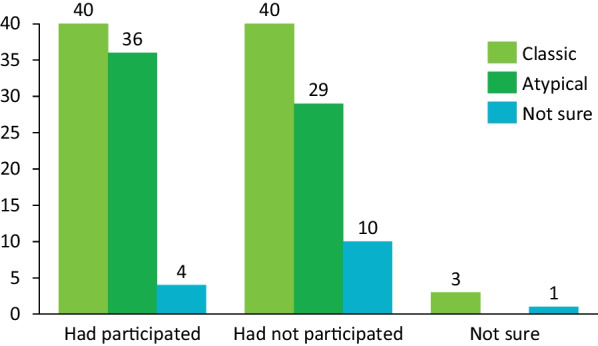
Clinical trial experience according to presenting PKAN phenotype. Respondents were grouped into classic (pink) and atypical (green) PKAN phenotype groups based on their reported age of onset (classic, < 10 years). Respondents who were unable to provide an age of onset were grouped as “not sure” (purple). PKAN = pantothenate kinase-associated neurodegeneration

Approximately half of the respondents (48%) indicated having taken part in a clinical trial or research study, and when considered by phenotype group, the proportions were similar groups (Fig. 5). Respondents who replied affirmatively were asked to describe the most difficult parts of participating in the trial. Most respondents reported that imaging assessments (eg, X-rays or MRIs) were the most difficult. Difficulty with imaging assessments was reportedly greater for those in the classic PKAN group; difficulty in holding a specific posture long enough to produce a clear image was noted. The reported difficulty with other assessments was related to the exertion necessary to complete assessments related to movement capabilities such as writing or walking.

**Fig. 6:**
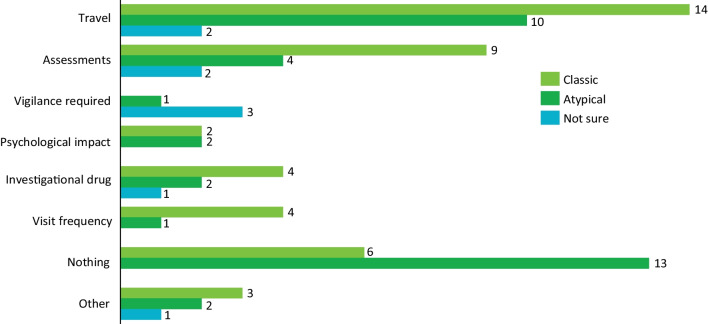
Difficulties related to clinical trial participation according to presenting PKAN phenotype. Respondents were grouped into classic (pink) and atypical (green) PKAN phenotype groups based on their reported age of onset (classic, < 10 years). Respondents who were unable to provide an age of onset were grouped as “not sure” (purple). The number of respondents reporting each category as most difficult was determined from the total number of respondents who answered affirmatively to having participated in a clinical trial. PKAN = pantothenate kinase-associated neurodegeneration

Outside of physical difficulties, travel to and from the trial site was also reported as being the most difficult aspect of participation in a trial (Fig. 6). Respondents were asked for methods they felt would improve the experience; most preferred less travel and improvements in the visit experience itself by reducing imaging and other assessments that required intense physical exertion (eg, writing and mobility tests). Respondents also expressed a desire for a more efficacious drug treatment.

## Discussion

This study used a strategy of engaging PAOs and patients with PKAN and their caregivers and families as research collaborators to assess the experiences and impact of PKAN from the patient community perspective. From a sample of 166 individuals, mostly parents of patients with PKAN, we found that our population could be distinguished using the putative classic versus atypical PKAN phenotype classifiers, with classic PKAN being defined here as an age of onset under 10 years. Similar to other studies [3, 7], the classic PKAN group defined here demonstrated the previously described characteristics of early onset and rapid progression, as well as initial symptoms of dystonia and visual impairment. The atypical PKAN group demonstrated a wider range of age of onset and initial symptoms of dystonia, parkinsonism, and speech difficulty. Among both groups, motor impairments were reported as main drivers of the large disease burden overall. Motor impairments were also reported as the greatest impediment to clinical trial participation, being hindered mostly by patients’ inability to travel easily to trial sites and by the physical difficulties associated with imaging assessments.

Because a better understanding of the expression of PKAN can improve trial design and accuracy of clinical research results, we sought to compare patient/caregiver perspectives between the classic and atypical PKAN phenotypes. Our findings support those of Marshall et al., [8, 9] who also reported that both phenotypes represent a large disease burden within the PKAN patient community . It is likely that this large burden is due to the neural targets of PKAN: brain areas dedicated to motor functions whose damage can make most of the activities of daily living difficult or impossible.

Our survey results add to the scientific and medical understanding of PKAN by identifying notable differences in the real-world experiences of patients with classic versus atypical PKAN. The categories are defined by age of onset, but we found that both the mean ages of onset and the ranges reported varied. The range of ages of onset in the classic PKAN group (0.1–9.5 years) spanned 9.4 years, whereas that in the atypical PKAN group (10–35 years) spanned 25 years; these ranges correspond reasonably well with those found in the literature [3, 7, 10], and the difference between them likely reflects the greater heterogeneity of the atypical PKAN classification. Respondents in both groups reported balance and gait problems (described as falls and reported “clumsiness”, respectively) as the first noticeable symptoms. However, compared with those in the atypical PKAN group, respondents in the classic PKAN group were more likely to report visual problems very early in addition to the initial motor problems; loss of ambulation in this group occurred roughly twice as often. Conversely, respondents in the atypical PKAN group were more likely to report cognitive or behavioral problems. The atypical PKAN group also reported more motor problems in addition to dystonia (eg, speech/swallowing problems or unusual limb movements) that were usually less severe, allowing many of these patients to retain some walking ability. One important caveat regarding these differences is their magnitude; the differences between groups in the initial symptom reports ranged from 10 to 16%, which is a distinguishable but modest difference given the size of the available sample. Thus, our results suggest that there is a valid distinction between classic and atypical PKAN phenotypes that includes considerable overlap between groups. The patient community expressed interest in exploring these differences further (López and Rintell, personal communication), suggesting that the distinction between phenotypes may become clearer as the database of patient community experiences grows.

The survey results indicated that patient experiences related to disease burden and management are mostly similar despite phenotypic differences. Both groups reported high levels of disease burden and relatively low quality of life. Respondents in both groups also noted that dystonia and parkinsonism were their most problematic symptoms, which suggests that motor impairments are the strongest driver of disease burden. This is supported by the survey results regarding disease management, in which respondents described how patients’ motor dysfunction increased disease burden. Patients reported difficulties in receiving medication, sometimes needing feeding tubes or other assistance. Medical care outside the home posed further problems, in that traveling to a doctor’s office or clinic usually required help from 1 or more caregivers; once there, the physical demands of neurologic tests, particularly imaging tests in which subjects must remain motionless, proved to be a large physical and emotional burden on both the patients and their caregivers.

The motor dysfunction in classic PKAN is often worse than that in atypical PKAN, and disease onset usually occurs before the age at which a healthy individual would be fully independent. Thus, classic PKAN would require larger time outlays by caregivers, or more caregivers, than atypical PKAN and likely represents a greater disease burden than the atypical phenotype [8].

Taken together, the survey results and clinical manifestations of PKAN suggest that even when accounting for the overlap of characteristics, there is a dichotomy between classic and atypical PKAN that extends beyond the age of onset and may include symptom severity and/or speed of disease progression. Interestingly, the survey results also suggested that disease management practices are generally similar regardless of phenotype. Further research is needed to determine whether this finding might represent evidence against the classic versus atypical classification scheme. More likely, it represents a significant unmet need for more efficacious treatments (ie, current management options provide at most modest symptom relief) [8, 9].

Notably, about half of the patients represented in the survey results had participated in clinical research. This was true for both the classic and atypical PKAN groups. To probe their experiences as trial participants, patients who had participated previously were asked to identify the greatest difficulties they faced in trial participation. The 2 most common responses, traveling to and from the site and the imaging tests that were a necessary part of the trial assessments, share a common source: PKAN-related motor dysfunction. The effect of loss of ambulation on traveling to and from the site is not surprising. However, the difficulty with imaging tests may be less clear until one considers that a patient experiencing dystonia and/or parkinsonism would require severe physical exertion to maintain a specific posture (particularly when also remaining as still as possible) for the length of time needed for the equipment to capture the image clearly. Thus, to improve participation and retention of patients with PKAN in clinical trials, every attempt should be made to reduce the number of motor-based assessments and simplify and shorten those that are necessary to assess therapeutic efficacy. Identifying alternatives to traveling to trial sites, such as using wearable monitors to allow home-based assessments, might also remove some of the roadblocks to trial participation experienced by these patients. Improving clinical trial design in these ways could expand the pool of participants and potentially reduce dropout, as well as potentially heighten the accuracy of the results.

To assess if PAO and patient community engagement is a viable research strategy that will produce accurate results reflective of the real world, we compared our results with those of other previously published studies. We found that the survey results compared favorably with the available literature on PKAN, even when our population was classified by phenotype (ie, classic or atypical PKAN). For example, Hayflick et al. found mean ± SD ages of onset of 3.4 ± 3.0 years and 13.7 ± 5.9 years for the classic and atypical phenotype groups, respectively, while other studies have reported similar median ages of onset [3, 7, 8, 10]. These values correspond well to those from this survey. Our age of onset ranges for each category were also similar to those reported in some studies [8, 10]. However, Li et al. [3] identified early- and late-onset cases in their 7-patient cohort but did not classify them separately, and Marshall et al. [8] grouped their patients in 4 disease severity groups. For the latter study, the wide range of ages of onset and similar median ages between severity groups led the authors to define PKAN phenotypes as a spectrum rather than a dichotomy, although their small sample size (n = 35) makes drawing such conclusions difficult [8].

The survey results also reflect the published literature in terms of disease manifestations and burden. Published data describe dystonia and gait abnormalities as the most common initial symptoms [3, 8] and neurologic motor and cognitive impairments and retinal degeneration as the most common clinical features of classic PKAN [2, 6, 7, 10]. For atypical PKAN, the data describe dystonia and gait disturbance, parkinsonism, and speech difficulty as the most common initial symptoms and speech difficulty, dystonia, and psychiatric and cognitive symptoms as the main clinical features [2, 6, 7, 10]. The results of this survey are in line with the published findings. The similarity of these fundamental aspects of PKAN is reflected by the similarity between the results of our survey and other studies assessing the disease burden and management efforts of patients with PKAN and their caregivers, which also show that disease burden is high and management strategies are only modestly effective and mostly limited to alleviating or mitigating symptoms [2, 8, 9, 11]. Overall, the results of our study are comparable with those of other published studies and suggest that collaborating with PAOs and their patient communities in PKAN research will produce accurate, meaningful findings.

This study had several limitations. The questions covered some, but not all, aspects of the disease, and the surveys themselves were limited to six languages. In addition, the needs for caregivers to fill out the survey and for the non-English language survey responses to be translated back to English conceivably could have introduced minor errors in accuracy. However, the use of grounded theory analysis (see Methods) should have minimized this.

## Conclusions

The results of this study laid a foundation for assessing PKAN and its impacts from the perspective of patients and their caregivers, and for addressing the unmet need to reduce the large disease burden and high medical utilization in this community. The involvement of international PAOs resulted in practical and relevant information about PKAN for patients and their families, as well as for clinicians and companies developing potential therapies for the disease. The survey identified motor impairments in PKAN as presenting the largest roadblocks to patient participation in clinical trials; thus, identifying alternatives to the frequent travel required and imaging procedures currently used in PKAN clinical trials could improve patient recruitment (and thus accuracy of results) in the future.

## Methods

A survey was developed to assess community perspectives on PKAN, and was distributed to patients with PKAN, their families, and/or their caregivers between November 2021 and January 2022. The survey was developed by creating an initial bank of multiple-choice and open-field questions. These were sent to NBIA PAOs in the United States, Poland, Germany, Spain, Italy, the Dominican Republic, and Switzerland, who edited and reviewed the question bank for clarity and appropriateness and returned their recommendations. The questions were then refined, and the review cycle repeated several more times to fine tune the questions for accuracy and functionality. A final set of 39 questions (27 multiple choice, and 12 open field; see Additional file 3 for the complete survey) were finalized and translated from English to Polish, German, Dutch, Italian, and Spanish, after which all 6 versions were uploaded to the SurveyPlanet™ online survey creation platform. The survey was submitted to the WCG IRB for approval; IRB approval of the study was waived because it only included survey-based interactions with subjects and provided adequate provisions for privacy and confidentiality of subject data. Participants provided informed consent for the use of their anonymized data for research.

Participants were recruited through the PAOs, who provided instructions on accessing and completing the survey, including information on the survey’s purpose, its provision of anonymity, and the expected time requirement. Participants were also told they did not have to answer every question to complete the survey. If the participant was a family member or caregiver assisting more than one patient with PKAN, they were instructed to complete separate surveys for each patient. Survey responses were translated back to English when necessary and compiled in Microsoft Excel™ for validation and analysis. For multiple-choice questions, statistical analysis was performed using descriptive statistics in Excel. For open-field questions, grounded theory analyses were performed [12] using both initial and focused coding approaches. Descriptive statistical analyses were performed and differences that met or exceeded 10% between groups were identified.

### Supplementary Information

**Additional file 1: Table S1.** Full List of Reported Medicines Used by Patients with PKAN.

**Additional file 2: Table S2.** Full List of Reported Wellness Products Used by Patients with PKAN.

**Additional file 3:** PKAN Patient Survey.

## Data Availability

Data not included here can be made available on request.

## Abbreviations

NBIA: Neurodegeneration with brain iron accumulation
MRI: Magnetic resonance imaging
PAO: Patient advocacy organization
PKAN: Pantothenate kinase-associated neurodegeneration
