# Supplementary material for: Patient and caregiver experiences with pantothenate kinase-associated neurodegeneration (PKAN): results from a patient community survey
Source: Orphanet J Rare Dis. 2023 Aug 31;18:257. doi: 10.1186/s13023-023-02869-1 (PMC10472673; doi:10.1186/s13023-023-02869-1)
Supplement: Supplementary file 1 — Additional file 1: Table S1. Full List of Reported Medicines Used by Patients with PKAN. [file 13023_2023_2869_MOESM1_ESM.pdf]

**Supplementary Table 1. Full List of Reported Medicines Used by Patients with PKAN**

| Class / Drug | Number of Mentions |
|---|---|
| ***Grand Total*** | ***532*** |
| **Anticonvulsants** | **146** |
| Clonazepam | 39 |
| Gabapentin | 27 |
| Diazepam | 23 |
| Phenobarbital | 12 |
| Valproate | 11 |
| Lorazepam | 8 |
| Pregabalin | 4 |
| Midazolam | 4 |
| Levetiracetam | 4 |
| Clobazam | 3 |
| Estazolam | 3 |
| Carbamazepine | 2 |
| Nitrazepam | 2 |
| Benzodiazepines (unspecified) | 1 |
| Temazepam | 1 |
| Bromazepam | 1 |
| Oxcarbazepine | 1 |
| **Antiparkinsonians** | **97** |
| Trihexyphenidyl | 59 |
| Levodopa-Carbidopa | 11 |
| Biperiden | 7 |
| Amantadine | 6 |
| Benserazide | 5 |
| Levodopa | 4 |
| Levodopa-Benserazide | 4 |
| Benzatropine | 1 |
| **Skeletal Muscle Relaxants** | **96** |
| Baclofen | 78 |
| Tizanidine | 18 |

| Class / Drug | Number of Mentions |
|---|---|
| **Antipsychotics** | **26** |
| Quetiapine | 8 |
| Haloperidol | 4 |
| Promazine | 4 |
| Pimozide | 2 |
| Chlorprothixene | 2 |
| Pipamperone | 1 |
| Risperidone | 1 |
| Aripiprazole | 1 |
| Tiapride | 1 |
| Olanzapine | 1 |
| Levomepromazine | 1 |
| **None** | **23** |
| None | 23 |
| **Analgesics** | **19** |
| Tramadol-Paracetamol | 8 |
| Morphine | 5 |
| Naproxen | 2 |
| Ibuprofen | 1 |
| Paracetamol | 1 |
| Analgesics(unspecified) | 1 |
| Methadone | 1 |
| **Iron Chelator** | **17** |
| Deferiprone | 17 |
| **Gastric Acid Secretion Inhibitors** | **13** |
| Omeprazole | 7 |
| Lansoprazole | 2 |
| Pantoprazole | 1 |
| Famotidine | 1 |
| Esomeprazole | 1 |
| heartburn med (unspecified) | 1 |

## Supplementary Table 1 (continued). Full List of Reported Medicines

| Class / Drug | Number of Mentions |
|---|---|
| **Non-CNS stimulant** | **13** |
| Clonidine | 12 |
| Guanfacine | 1 |
| **Antidepressants** | **10** |
| Trazodone | 3 |
| Fluvoxamine | 2 |
| Sertraline | 1 |
| Paroxetine | 1 |
| Escitalopram | 1 |
| Bupropion | 1 |
| Mirtazapine | 1 |
| **Antihistamine H1** | **9** |
| Diphenhydramine | 4 |
| Cetirizine | 3 |
| Meclizine + Pyridoxine | 1 |
| Hydroxyzine | 1 |
| **Antichorea** | **8** |
| Tetrabenazine | 8 |
| **Trial - 4'-phosphopantetheine compoumd** | **8** |
| CoA-Z | 8 |
| **Laxative** | **7** |
| Movicol | 3 |
| Senna | 1 |
| Naturalex | 1 |
| Miralax | 1 |
| Docusate Sodium | 1 |
| **Antiepileptics** | **5** |
| Lacosamide | 3 |
| Phenytoin | 1 |
| Antiepilepticss (unspecified) | 1 |

| Class / Drug | Number of Mentions |
|---|---|
| **Hypnotics** | **3** |
| Chloral hydrate | 2 |
| Chloropromazine | 1 |
| **Retinoids** | **3** |
| Tretinoin | 3 |
| **Neuromuscular Blocker** | **3** |
| Botulinum Toxin | 3 |
| **(blank)** | **3** |
| Medical marijuana | 1 |
| Lurisin | 1 |
| Therapeutic cannabis | 1 |
| **Hormones** | **2** |
| Levothyroxine | 2 |
| **Cognition Enhancer** | **2** |
| Piracetam | 2 |
| **Cholinergic** | **2** |
| Bethanechol | 2 |
| **CNS Stimulant** | **2** |
| Lisdexamfetamine | 1 |
| Atomoxetine | 1 |
| **Antihypertensives** | **2** |
| Lisinopril | 1 |
| Amlodipine | 1 |
| **Beta-blocker (Cardioselective)** | **2** |
| Metoprolol | 1 |
| Bisoprolol | 1 |
| **Beta-3 adrenergic agonists** | **1** |
| Mirabegron | 1 |
| **Alpha Blocker** | **1** |
| Tamsulosin | 1 |
| **Contraceptive** | **1** |
| Contraceptive | 1 |

## Supplementary Table 1 (continued). Full List of Reported Medicines

| Class / Drug | Number of Mentions |
|---|---|
| **Anxiolytics** | **1** |
| Anxiolytics (unspecified) | 1 |
| **Antispasmodics** | **1** |
| Oxybutynin | 1 |
| **Antiemetics** | **1** |
| Domperidone | 1 |
| **Stimulant Purgative** | **1** |
| Prucalopride | 1 |

| Class / Drug | Number of Mentions |
|---|---|
| **Diabetes meds** | **1** |
| Diabetes meds (unspecified) | 1 |
| **Statin** | **1** |
| Pravastatin | 1 |
| **Leukotriene Antagonist** | **1** |
| Montelukast | 1 |
| **Anticholinesterases** | **1** |
| Pyridostigmine | 1 |
