# Supplementary material for: Patient and caregiver experiences with pantothenate kinase-associated neurodegeneration (PKAN): results from a patient community survey
Source: Orphanet J Rare Dis. 2023 Aug 31;18:257. doi: 10.1186/s13023-023-02869-1 (PMC10472673; doi:10.1186/s13023-023-02869-1)
Supplement: Supplementary file 2 — Additional file 2: Table S2. Full List of Reported Wellness Products Used by Patients with PKAN. [file 13023_2023_2869_MOESM2_ESM.pdf]

Supplementary Table 2. Full List of Reported Wellness Products Used by Patients with PKAN

Product	Number of Mentions	Product	Number of Mentions
Grand Total	*436*	Probiotics	4
Vitamin B	83	THC/CBD oil	4
Fatty acids	56	Zinc	3
Vitamin D	50	Resveratrol	3
CoQ10	32	Potassium	3
Pantethine	30	Vitamin K	2
None	30	ALA	2
Multivitamins	22	Elderberry	2
Diet	21	Coenzyme A	2
Vitamin C	20	Diet	1
Vitamin E	15	Aloe	1
Magnesium	10	Chromium	1
Melatonin	8	Cranberry Caps	1
Iron	6	Other	1
L-carnitine	6	Glutathione	1
Vitamin A	5	Minerals	1
GABA	4	NAD+	1
Calcium	4	NAC	1
