# Supplementary material for: Patient and caregiver experiences with pantothenate kinase-associated neurodegeneration (PKAN): results from a patient community survey
Source: Orphanet J Rare Dis. 2023 Aug 31;18:257. doi: 10.1186/s13023-023-02869-1 (PMC10472673; doi:10.1186/s13023-023-02869-1)
Supplement: Supplementary file 3 — Additional file 3: PKAN Patient Survey. [file 13023_2023_2869_MOESM3_ESM.pdf]

The following survey was also translated into Polish, German, Dutch, Italian and Spanish

PKAN Patient Survey

CoA Therapeutics is developing an investigational treatment of pantothenate kinase associated neurodegeneration (PKAN). The aim of this survey is to learn from individuals affected by PKAN about what they consider greatest burdens of disease, to guide development of a future clinical trial.

Completing this survey is entirely voluntary, you do not have to complete this survey if you do not want to.

It takes about 30-45 minutes to complete this survey. Please complete it at a time when you can do so from start to finish. If you need to step away from the survey before you finish, please leave the browser open. Closing the browser before completing it will erase the answers you entered.

If possible, please complete **one survey for each person affected by PKAN** in your family.

Anonymity

The survey is completely anonymous, you will not be asked to disclose any information that could identify you like names or addresses. Please do not include any information that could identify you or anyone else.

We thank you very much for your time!

1. Are you: Mandatory Question
   - o An individual with PKAN
   - o A parent of an individual with PKAN
   - o Other caregiver of an individual with PKAN

2. What is the current age of the individual with PKAN?
   Open text: ___________

3. Which country does the individual with PKAN live in? Mandatory Question
   - o Netherlands
   - o Italy
   - o Germany
   - o Poland
   - o Spain
   - o Switzerland
   - o US
   - o UK
   - o Other: Open text: ___________

4. At what age did the individual with PKAN first experience PKAN-related symptoms?
   - Open text: ___________
   - I'm not sure

5. Thinking back, what was the very first PKAN-related symptom(s) the individual experienced? (Multiple selections allowed)
   - o Muscle problems (Muscle cramping, unusual movements, unusual postures)
   - o Problems with balance/falls
   - o Problems with motor skills
   - o Speech/swallowing problems
   - o Vision problems (night blindness, peripheral vision problems)
   - o Behavioral, emotional or cognitive challenges
   - o Other

6. At what age did the individual receive a PKAN diagnosis?
   - o Open text ____
   - o Not sure

7. Did a genetic test confirm the PKAN diagnosis?
   - o Yes
   - o No
   - o Not sure
   - o Other: _________________

8. Does the individual with PKAN understand verbal questions and instructions?
   - o Yes
   - o No
   - o Sometimes
   - o Not sure
   - o Not applicable (e.g. too young to be expected to understand)

9. The individual with PKAN:
   - o Can say a few words
   - o Can say complete sentences
   - o Can't speak at all
   - o Other: _________________

10. How does the individual with PKAN generally communicate with others? (*select all that apply)*
    - o Verbally (e.g. with words/sounds)
    - o Non-verbally (e.g. with body language/movements)
    - o Written
    - o Communication assistance device
    - o Other: ___________

11. The individual with PKAN:
    - o Can read some words
    - o Can read complete sentences
    - o Can't read at all
    - o Other: _________________

12. The individual with PKAN:
    - o Can write some words by hand
    - o Can write complete sentences by hand
    - o Can't write by hand at all
    - o Other: ______________

13. The individual with PKAN:
    - o Can walk independently
    - o Can walk if assisted by someone or a device, orthesis, sticks, walker
    - o Can't walk at all
    - o Other:___________________

14. The individual with PKAN:
    - o Can sit independently
    - o Can sit assisted by someone or with support devices
    - o Can't sit at all
    - o Other:___________________

15. Does the individual with PKAN have vision problems such as night blindness, loss of (peripheral) vision, problems with glaring light, or other vision issues?
    - o Yes
    - o No
    - o Not sure

16. What top three (3) PKAN symptoms are the hardest/most challenging for the individual with PKAN to live with?
    Open field:_______________________

17. What are the top three (3) symptoms of PKAN that you would hope could be improved by a new treatment?
    - o Open field:

18. What medications does the individual with PKAN take on a routine basis?
    - o Open field:_____________

19. What vitamins/supplements/wellness products does the individual with PKAN take on a routine basis?
    - o Open field:________________

20. Does the individual with PKAN: (Select all that apply)
    - o get Botox injections regularly?
    - o have a Baclofen pump implanted?
    - o have a tracheostomy tube ("trach")?
    - o use non-invasive breathing assistance, such as Bilevel positive airway pressure (BiPAP)?
    - o None of the above

21. Does the individual with PKAN have a deep brain stimulation device implanted?
    - o Yes
    - o No
    - o Prefer not to say

If YES (go to Q 22-23) ; If NO (go to Q24)

22. At what age was their deep brain stimulation device implanted?
    - o Open field:________________

23. Is their deep brain stimulation device rechargeable?
    - o Yes
    - o No
    - o Not sure

24. What medical assessments (such as lab tests, questionnaires, physical tests, etc.) are the most difficult?
    - o Blood draws
    - o Urine/stool samples
    - o physical tests (e.g. movement, strength, reflex tests)
    - o Imaging (e.g. X-rays, MRIs, CT scans)
    - o Filling out questionnaires
    - o Other: ___________

25. Why do you rate this medical assessment as the most difficult?
    - o Open field:________________

Formulation Questions

26. Does the individual with PKAN use a feeding tube?
    - Yes
    - No

If YES go to Q27-28, then Q33 (do not ask by mouth questions); if NO go to Q29

27. What kind of feeding tube does the individual with PKAN have?
    - o Gastrostomy tube
    - o J-PEG
    - o Nasogastric tube
    - o Other, please specify: ______________

28. Does the individual with PKAN use a different method for taking medications versus feeding?
    - o Yes, by mouth ((if selected go to Q29))
    - o Yes, different (please specify): ____________________
    - o No, the same

29. Which of the following types of medications **can** the individual with PKAN **take by mouth**? Select all that apply. Please note, a pill could be a tablet or capsule.
    - o Small pills (less than 8 mm long)
    - o Medium pills (8-12 mm long)
    - o Large pills (greater than 12 mm long)
    - o Liquids
    - o Chewables
    - o Granules (similar to a powder, can be sprinkled on food)
    - o Tablet placed on or under the tongue that mixes with the saliva
    - o None of the above

30. Does the individual with PKAN **prefer** any of the following types of medications to take **by mouth**? Select all that apply. Please note, a pill could be a tablet or capsule.
    - o Small pills (less than 8 mm long)
    - o Medium pills (8-12 mm long)
    - o Large pills (greater than 12 mm long)
    - o Liquids
    - o Chewables
    - o Granules (similar to a powder, can be sprinkled on food)
    - o Tablet placed on or under the tongue that mixes with the saliva
    - o None of the above

31. Does the individual with PKAN have any **flavor preference** that makes it easier for them to take a medication by mouth? Please specify:
    - o No preference
    - o Unflavored
    - o Sweet flavored
    - o Specific flavor (Strawberry, Raspberry, Cherry, Grape, Banana, Mint, etc.)
    - o Other
    - o Not sure

32. Which of the following **ways of taking medication by mouth** is **most convenient** for the individual with PKAN or the caregiver?
   - o Swallowing whole tablets/capsules (e.g. with a glass of water)
   - o Breaking up tablets into smaller parts before swallowing (e.g. with a glass of water)
   - o Crushing tablets to a powder or opening capsules to extract the powder and mix it with liquid or food
   - o None of the above
   - o Other ______________

33. Does the individual with PKAN have any dietary restrictions, food allergies or drug allergies? Please describe.
   - o Open field ________

Clinical Trial Questions

34. Has the individual with PKAN ever participated in a clinical trial?
   - o Yes
   - o No
   - o Not sure

If YES go to Q35-36; If NO go to Q37

35. What were the hardest things about taking part in the trial?
   Open field:__________________

36. What could have improved the experience of participating in the clinical trial?
   Open field:____________________

Questions For a Future Trial

37. Which methods of transport are you able to use <u>when traveling with the individual with PKAN</u>:
   - o Public transit (e.g. metro)
   - o Car (sitting)
   - o Car (lying down)
   - o Train
   - o Plane
   - o Other
   - o None of the above

38. When the individual with PKAN travels, how many caregivers usually accompany them?
   - o One caregiver
   - o Two caregivers
   - o Other (please specify): __________
   - o None

39. Taking part in clinical trials usually involves traveling to a specific medical center for multiple visits that require physician assessment. If there were a clinical trial you were interested in, how far would you be able/willing to travel (assuming the cost of travel was reimbursed to you and translation services/support was available):
   - o Within my city/region
   - o Outside of my city/region, but in my country
   - o Outside of my country
   - o Other
